# Characteristics of the delayed respiratory syncytial virus epidemic, 2020/2021, Rhône Loire, France

**DOI:** 10.2807/1560-7917.ES.2021.26.29.2100630

**Published:** 2021-07-22

**Authors:** Jean-Sebastien Casalegno, Dominique Ploin, Aymeric Cantais, Elsa Masson, Emilie Bard, Martine Valette, Remi Fanget, Sandrine Couray Targe, Anne-Florence Myar-Dury, Muriel Doret-Dion, Mona Massoud, Gregory Queromes, Philippe Vanhems, Olivier Claris, Marine Butin, Sylvie Pillet, Florence Ader, Sylvie Bin, Alexandre Gaymard, Bruno Lina, Florence Morfin, VRS study group in Lyon, Etienne Javouhey, Yves Gillet, VRS study group in Lyon, VRS study group in Lyon, Antoine Ouziel, Jean-claude Tardy, Pascal Gaucherand, Luc Panetta, Jerome Massardier, Stephanie Polazzi, Antoine Duclos, Mehdi Benchaib, Regine Cartier, Marine Jourdain, Michelle Ottmann, Rolf Kramer, Sylvie Fiorini, Nathalie Rivat, Yahia Mekki, Julie Fort-Jacquier, Maud-Catherine Barral, Vey Noelie, Julie Haesebaert, Come Horvat, Leo Vidoni, Jean-Marc Reynes, Jean-Francois Eleouet, Laurence Josset, Matthieu Receveur

**Affiliations:** 1 Hospices Civils de Lyon, Hôpital de la Croix-Rousse, Centre de Biologie Nord, Institut des Agents Infectieux, Laboratoire de Virologie, Lyon, France; 2 Centre national de référence des virus des infections respiratoires (dont la grippe), Hôpital de la Croix-Rousse, Lyon, France; 3 Centre International de Recherche en Infectiologie (CIRI), Laboratoire de Virologie et Pathologie Humaine - VirPath Team, INSERM U1111, CNRS UMR5308, École Normale Supérieure de Lyon, Lyon, France; 4 Université Claude Bernard Lyon 1, Lyon, France; 5 Hospices Civils de Lyon, Hôpital Femme Mère Enfant, Service de Réanimation Pédiatrique et d’Accueil des Urgences, Bron, France; 6 Centre Hospitalier Universitaire de Saint-Étienne, Service des Urgences Pédiatriques, Saint-Priest-en-Jarez, France; 7 Groupe Immunité des Muqueuses et Agents Pathogènes (GIMAP) EA-3064, Faculté de Médecine de Saint-Etienne, Campus Santé-Innovations de Saint-Etienne, Saint-Priest-en-Jarez, France; 8 Hospices Civils de Lyon, Département d’Information Médicale, Lyon, France; 9 Hospices Civils de Lyon, Service de Gynécologie-Obstétrique, Hôpital Femme-Mère-Enfant, Bron, France; 10 Hospices Civils de Lyon, Centre Hospitalier Édouard Herriot, Service Hygiène, Épidémiologie et Prévention, Lyon, France; 11 Centre International de Recherche en Infectiologie (CIRI), Public Health, Epidemiology and Evolutionary Ecology of Infectious Diseases (PHE3ID) INSERM U1111, CNRS UMR5308, École Normale Supérieure de Lyon, Lyon, France; 12 Hospices Civils de Lyon, Hôpital Femme-Mère-Enfant, Service de Néonatologie et de Réanimation Néonatale, Bron, France; 13 Hospices Civils de Lyon, Pôle IMER, Unité de Recherche Clinique, Lyon, France; 14 Centre Hospitalier Universitaire de Saint-Étienne, Laboratoire de Virologie, Saint-Priest-en-Jarez, France; 15 Hospices Civils de Lyon, Hôpital de la Croix-Rousse, Service des Maladies Infectieuses et Tropicales, Lyon, France; 16 Additional members of VRS study group in Lyon who contributed to data collection are listed at the end of this article.

**Keywords:** RSV, bronchiolitis, non-pharmacological interventions, pharmacological interventions, COVID-19, SARS-CoV-2, SARI, disease burden

## Abstract

The Rhône-Loire metropolitan areas’ 2020/21 respiratory syncytial virus (RSV) epidemic was delayed following the implementation of non-pharmaceutical interventions (NPI), compared with previous seasons. Very severe lower respiratory tract infection incidence among infants ≤ 3 months decreased twofold, the proportion of cases among children aged > 3 months to 5 years increased, and cases among adults > 65 years were markedly reduced. NPI appeared to reduce the RSV burden among at-risk groups, and should be promoted to minimise impact of future RSV outbreaks.

The emergence of coronavirus disease (COVID-19) has triggered a wide-scale implementation of non-pharmaceutical interventions (NPI) including physical distancing, school closures, travel restrictions, and the use of masks in public spaces [1]. These preventive public health measures have impacted the circulation of respiratory syncytial virus (RSV) as demonstrated by inter-seasonal RSV epidemics in several southern hemisphere countries [2-4] and late-season RSV outbreaks in several European countries [5-7]. Here, we describe the age characteristics and the very severe lower respiratory tract infection (VS-LRTI) incidence of this late RSV epidemic in the Rhône-Loire metropolitan areas in France.

## Detection of respiratory syncytial virus cases

We prospectively collected laboratory data from the university hospitals of Lyon and Saint-Etienne in the Rhône-Loire metropolitan areas with ca 2 million inhabitants, from September 2018 to May 2021 [7]. All respiratory samples (nasopharyngeal aspirates, nasal/throat/oral swabs, tracheobronchial aspirates, and bronchoalveolar lavages) taken from patients with respiratory tract infection (upper or lower/mild or severe) and received by the virology laboratories from in- and outpatients were tested for RSV and included in our analysis.

An RSV case was defined as any laboratory-confirmed RSV infection detected by real-time reverse transcriptase (RT)-PCR during the study period. As SARS-CoV-2 and RSV co-detections are rare events [8], they were not further investigated in the present study.

## Calculation of hospitalisation rates and incidence of very severe and severe cases

In order to calculate hospitalisation rates, we collected data from children born at the Hospices Civils de Lyon over three consecutive years (1 June 2018–01 April 2021) whose parents are living in the Métropole de Lyon (around 1.4 million inhabitants). From this birth cohort (Lyon cohort), hospitalised cases with confirmed RSV infection during the first year of life were identified using the diagnostic laboratory database on three consecutive RSV seasons (2018/19; 2019/20 and 2020/21 between 1 September and May 2021). VS-LRTI was defined as a laboratory-confirmed RSV infection leading to hospitalisation occurring during the first year of life, and meeting the World Health Organization case definition (SpO_2_ < 90%, inability to feed) [9]. The incidence of severe RSV cases was estimated per 1,000 births as previously described [10]. To take into account the observed 3-month delay in the 2020/21 RSV seasonal epidemic, births between 1 April 2020 and 1 April 2021 were considered, whereas children born between 1 January 2019 and 31 December 2019 were considered for the analysis of 2019/20 RSV seasonal epidemic.

## Delayed seasonal epidemic

**Figure 1:**
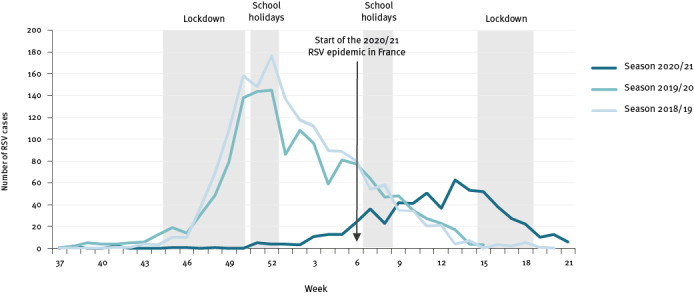
Number of laboratory-confirmed respiratory syncytial virus cases by week, university hospitals of Lyon and Saint-Etienne, France, epidemiological seasons 2018/19−2020/21

In the Rhône and Loire metropolitan areas, the RSV epidemic has a seasonal pattern: the first cases are usually detected at week 41, followed by a peak of cases around week 51 [7]. In 2020, the first RSV cases of the 2020/21 season were detected at week 38, and cases were then detected on weekly basis below the epidemic threshold from week 51 2020 to week 5 2021. In week 5 2021, the RSV epidemic started in the Île de France region, comprising the city of Paris, while the number of RSV cases started to gradually increase in Rhône-Loire [6]. The RSV epidemic finally started in the Rhône-Loire metropolitan areas in week 10 2021 with a 4-month delay and no timely correlation with any major change in the NPI strategy [11]. The weekly number of cases in the region decreased in week 16 2021 coinciding with the start of the third French lockdown including a 3-week school closure during weeks 14 to 16. The end of the 2020/21 RSV seasonal epidemic was declared in Rhône-Loire in week 16 2021 (Figure 1), however, RSV cases were still detected in this region at the end of this survey (week 21).

RSV: respiratory syncytial virus.

Grey areas represent non-pharmaceutical interventions and events during the 2020/21 season (lockdown, school holidays and closure). The epidemic dynamics are similar for seasons 2018/19 and 2019/20 (darker blue lines) and delayed for the 2020/21 season (light blue line).

Overall, 622 cases were detected in 2020/21 compared with 1,373 in 2019/20 and 1,420 in 2018/19 which represents a 2.2 mean decrease.

Subtyping of 132 of all 167 RSV grown in culture (20%) in Rhône-Loire in 2020/21, indicated a predominant RSV-A epidemic (119 RSV-A, 13 RSV-B).

## Atypical age distribution

**Figure 2:**
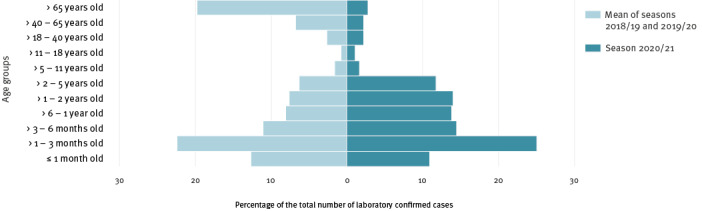
Age pyramid of respiratory syncytial virus cases by epidemiological season, university hospitals of Lyon and Saint-Etienne, France, epidemiological seasons 2018/19−2020/21^a^

We compared the age of RSV cases between the seasons 2020/21 (622 cases), 2019/20 (1,373 cases), and 2018/19 (1,420 cases). The age distribution is usually remarkably similar from one epidemic season to another (Figure 1) and U-shaped with a higher proportion of cases below 6 months and over 65 years of age. The notable features of the 2020/21 season were a decreased proportion of cases aged over 65 years of age and an increased proportion of cases in children aged over 3 months and up to 5 years (Figure 2B). The change in proportions in the older age group cannot be fully explained by changes in testing; we observed among cases aged 65 years and older a fourfold reduction in the number of RT-PCR tests in 2020/21 (n = 1,646) compared with 2019/20 (n = 6,763) while there was a 10-fold reduction in the number of cases (2020/21: n = 17; 2019/20: n = 238).

RSV: respiratory syncytial virus.

^a^ Mean of seasons 2018/19 and 2019/20 compared with season 2020/2021.

The number of cases is expressed as a percentage of the total number of laboratory-confirmed RSV cases per season (n = 2,793 for 2019/20 and 2018/19 and n = 622 for 2020/21).

## Reduced incidence of very severe lower respiratory tract infection in early infancy

As this unusual case distribution for the 2020/21 RSV season may result from changes in testing strategy or from a decrease in the number of cases among the at-risk groups, we compared the incidence of VS-LRTI with the previous 2019/20 season in the Lyon cohort (Table). The VS-LRTI incidence rate in under 1 year-olds was significantly lower in 2020/21 (8.8/1,000, n = 77/8,728, 95% confidence interval (CI): 6.0−10.0) compared with 2019/20 (13.6/1,000, n = 124/9,127, 95%CI: 11.0−16.0). The relative risk of VS-LRTI incidence was 1.5-fold lower (95%CI: 1.2−2.1) during the first year of life and twofold lower (95%CI: 1.4−2.9) during the first 3 months of life in the 2020 birth cohort compared with the 2019 one. Contrary to the sharp decrease observed in the at-risk groups, the absolute number of VS-LRTI among infants aged over 3 months and up to 1 year was stable (32 for 2020 birth cohort vs 29 for 2019 birth cohort).

**Table:** Incidence of very severe lower respiratory tract infection in the Lyon cohort^a^ by season, university hospital of Lyon, France, epidemiological seasons 2019/20−2020/21

Incidence and relative risk reduction by age group	Season 2019/20	Season 2020/21
	Births from 1 January to 31 December 2020	Births from 1 April 2020 to April 2021
Incidence of laboratory-confirmed RSV hospitalisation for VS-LRTI < 1 year of age	13.6/1,000 (95% CI: 11.0−16.0)	8.8/1,000 (95% CI: 6.0−10.0)
RR reduction of the incidence of hospitalisation for VS-LRTI < 1 year of age	1.5-fold lower (95% CI: 1.2−2.1)	
Incidence of laboratory-confirmed RSV hospitalisation for VS-LRTI ≤3 months of age	10.4/1,000 (95% CI: 8.0−13.0)	5.2/1,000 (95% CI 4.0−7.0)
RR reduction of the incidence of hospitalisation for VS-LRTI ≤3 months of age	2.0-fold lower (95% CI: 1.4−2.9)	
Total number of VS-LRTI case in HCL cohort aged over 3 months up to 1 year	29	32

CI: confidence interval; HCL: Hospices Civils de Lyon; RSV: respiratory syncytial virus; RR: relative risk; VS-LRTI: very severe lower respiratory tract infection.

^a^ Children born at the Hospices Civils de Lyon over three consecutive years (1 June 2018–01 April 2021) whose parents are living in the Métropole de Lyon.

To take into account the observed 3-month delay in the 2020/21 RSV epidemic, for this season, only births from 1 April 2020 to 1 April 2021 were considered.

## Ethical statement

This study has been approved by the Review Board of university hospitals of Lyon and is registered at ClinicalTrials.gov with identifier NTC04944160.

## Discussion

We describe a delayed 2020/21 RSV season with atypical features such as a reduced incidence of VS-LRTI among infants aged 3 months or less, a sharp reduction in the number of cases aged over 65 years, and in contrast, an increased proportion of cases detected among children aged over 3 months and up to 5-years, and a relative increase in the number of VS-LRTI cases among infants between 3 months and up to 1 year of age.

New RSV lineages could be introduced much later from the southern hemisphere because of the combined effect of border controls and the delay of the 2019/20 RSV outbreak in the southern hemisphere [12]. However, there has been no obvious change in COVID-19 NPI measures since September 2020 that would explain the seasonal epidemic start observed here from week 3 to 6 2021. At that time, face masks were mandatory for the population above 6 years of age both in indoor and outdoor settings, in public spaces and at work places, while primary schools and shops were open with hygiene/preventive protocols in place, high schools were only partially open, working from home was strongly recommended, restaurants were closed, and a national curfew at 7p.m. was enforced. We hypothesise that either those NPI did not fully prevent RSV from circulating among school-age children while schools were open, or there was a decreased compliance during this period regarding some or several of those NPI. This observation highlights the risk of a RSV outbreak within a population experiencing low levels of RSV circulation and despite the implementation of NPI in case of opened schools [13].

The twofold decrease in incidence among infants aged 3-months and less was likely due to the decreased exposure of newborns to RSV, which may be related to both the overall reduced size of the RSV epidemic and behavioural changes towards newborns. Simple prevention recommendations such as hand washing and avoiding close contact with sick people should be strongly and timely promoted to future parents, and may constitute one of the main options to reduce the burden of the next RSV season. The sharp decrease in the number of detected cases among people aged over 65 years is surprising, and cannot be fully explained by the decreased number of combined influenza/RSV RT-PCR tests. Therefore, NPI implementation targeting elderly people and reduced intergenerational contacts appear to have contributed majorly to reducing infections in this age group.

Altogether, the increased proportion of RSV cases among infants between 3 months and 1 year of age and the stable number of hospitalised VS-LRTI cases among infants between 3 months and 1 year of age were in sharp contrast with the RSV burden reduction in the at-risk groups. The increase in the median age of Australian paediatric cases was a main feature of this 2020 delayed epidemic compared with previous years [2]. Our study presents many strengths related to the sample size considered (metropolitan population coverage), the combined use of data from two centres, and the use of a birth cohort focusing on hospitalised VS-LRTI cases, providing confidence that the testing strategy was unlikely to have affected our results [5]. This relative increase is, therefore, more likely related to an age-independent VS-LRTI-related phenotype developing at an older age because of the delayed epidemic. The frequency of such events among at-risk children is probably low, but the number of cases might have accumulated over time since the last epidemic offset. The age-independent RSV-related VS-LRTI requires further monitoring and investigation, as the longer the non-exposition period, the higher the final number of cumulative cases, which may add to the classical age-related phenotype i.e. no risk factor and infections occurring below the age of 3 months.

One main limitation of this study lies in the lack of power that did not allow us to exclude a significant increase in the VS-LRTI incidence among infants over 3 months and up to 1 year of age. Further investigations on the delayed RSV epidemics are required to explore the increased risk of VS-LRTI among infants over 3 months of age and to provide a clinical description of these age-independent VS-LRTI-related phenotypes. One other main limitation is that we were not able to consider more RSV seasons in VS-LRTI incidence calculation. However, the 2019/20 incidence of VS-LRTI is in the range of expected values for a mean RSV season. This limitation is thus unlikely to have influenced the results [10,14]. Finally, we did not investigate climate-driven factors such as specific humidity, precipitation, and temperature that might have played a role in shaping this late RSV dynamic [15].

### Conclusions

Our analysis highlights the risk of an RSV outbreak within a population experiencing low levels of RSV circulation and despite the implementation of NPI, in cases where schools are not closed. We also reported differences in the age groups affected with, on the one hand, a reduced RSV burden among very young infants aged 3 months or less and adults aged over 65 years old, and on the other hand, a relative increase among pre-school age children. Stricter adhesion to RSV prevention measures generally recommended by public health institutes such as washing hands, avoiding close contact with sick people and covering the mouth when coughing should be promoted to the families of newborns until the baby is at least 3 months old. A combination with passive immunisation for the at-risk newborns i.e. preterm babies and those with underlying pulmonary diseases should be also considered to minimise the potential impact of future RSV outbreaks on healthcare systems [16].
